# Assessing Movements between Freshwater and Saltwater by Brown Trout (*Salmo trutta* L.) Based on Otolith Microchemistry

**DOI:** 10.3390/ani14142116

**Published:** 2024-07-20

**Authors:** Magdalena Andersson, Bror Jonsson, Olle Calles, Larry Greenberg

**Affiliations:** 1 River Ecology and Management Research Group, Department of Environmental and Life Sciences, Karlstad University, 651 88 Karlstad, Sweden; magdalena.andersson@orebro.se (M.A.); bror.jonsson@nina.no (B.J.); olle.calles@kau.se (O.C.); 2 Norwegian Institute for Nature Research, 0855 Oslo, Norway

**Keywords:** precocious migrants, life history, salmonid, Baltic Sea, phenotypic variation, strontium, calcium, migration

## Abstract

**Simple Summary:**

Otoliths, which are calcium carbonate structures found in the inner ears of fish, can be used to describe the use of freshwater and saltwater habitats during the lifespan of fish. This is performed by looking at the ratio of strontium to calcium along the radius of an otolith, where a high ratio indicates the use of saltwater and a low ratio indicates the use of freshwater. We studied brown trout (*Salmo trutta* L.) from the Swedish River Emån. We found that most brown trout, as expected, spent the first 1–2 years of their lives in freshwater before migrating to the Baltic Sea. We also found that 13% spent considerably less than one year in the river, which was not expected based on classical life history patterns for brown trout. In addition, we found that brown trout, which ranged from 3 to 6 years of age, returned to freshwater 2.3 times, and the amount of time spent in freshwater after hatching was negatively related to the number of visits to freshwater. Previous studies have explained that precocial migration occurs when there is a risk of drought. This explanation cannot apply to the permanently flowing River Emån.

**Abstract:**

By analyzing otolith microchemistry, we examined the use of freshwater and marine environments by brown trout *Salmo trutta* L. that spawn in the Swedish River Emån and migrate to the Baltic Sea. We estimated the time juveniles spent in freshwater and the number of times the fish returned to freshwater, presumably to spawn. Twenty-six percent of the fish migrated to sea by 1 year of age. However, 13% spent less than one year in the river. Most brown trout (48%) migrated to the sea between 1 and 2 years of age. On average, brown trout, which averaged 4.4 years in age (range 3–6 years), returned to freshwater 2.3 times, and there was an inverse relationship between time spent in freshwater after hatching and the number of visits to freshwater. Our results do not support the classical life history pattern, where brown trout spend one or more years in freshwater before migrating to the sea. Here, we found evidence that part of the population leaves freshwater during their first year. While the cause for precocial migration in the River Emån is not known, our results from this permanently flowing river do not support the idea proposed for other Baltic Sea populations, where the risk of drought has been suggested to be the cause.

## 1. Introduction

The life cycle of most salmonids is diverse. Many of the species consist of migratory, non-migratory, and partial migratory populations. Even the destination of their migration is variable, with some populations migrating to rivers, whereas others migrate to lakes, estuaries, or the sea [1,2,3]. The typical pattern for one salmonid species, the brown trout (*Salmo trutta* L.), is that it spends the first year or years of its life in its natal stream, whilst habitats used later in life are variable. Hence, there is extensive phenotypic variation in the age at which brown trout migrate as well as in how long they remain in the alternate habitat before returning to their natal river to reproduce [4].

Many populations of brown trout are anadromous and after having smolted (a physiological transformation preparing immatures at ages between 1 and 5 years for a pelagic lifestyle in saltwater (cf. [5])), immatures leave freshwater to feed in brackish water or the sea [6]. They typically feed in fjords and coastal waters, but more seldomly, they move into the open ocean to feed [7]. In the Baltic Sea, anadromous brown trout stay in brackish water after they leave their home river and remain until they attain sexual maturity and return for spawning [8]. In the Atlantic Ocean, where salinity is higher, not only sexually mature brown trout return to the river, but also immature individuals may return after having fed at sea. In the latter case, immature individuals spend the winter in a suitable freshwater habitat before returning to the sea for feeding in the subsequent spring [9]. Being iteroparous, brown trout may perform spawning migrations several times during their lifetime, although the cost of reproduction is high, and many adults die after having spawned only once [10]. Large brown trout are typically repeat spawners.

The classical description of anadromous brown trout spending its first year or more of life in its natal stream before smolting and migrating to the sea has been challenged. In the Baltic Sea, for instance, brown trout have been shown to enter the sea during their first summer. During periods of summer drought, Ref. [11] reported that young-of-the-year brown trout originating from a small creek on the Swedish island of Gotland were observed as they emigrated from their nursery stream into the Baltic Sea, and a similar phenomenon may occur in some coastal streams on the Swedish mainland [12]. Similarly, a study from Estonia reported that fry of anadromous brown trout enter the Baltic Sea shortly after the yolk sac is absorbed [13].

Phenotypic variation in the origin of fish (freshwater vs. saltwater), the age at which they leave freshwater, and how often they return from the sea can be studied using microchemical analysis of otoliths [12,13,14,15,16,17,18,19]. For instance, the relative concentrations of strontium (Sr) and calcium (Ca) in otoliths reflect the salinity of the water [20], and thus, movement patterns of anadromous fish between freshwater and saltwater can be elucidated by chemical analysis of otoliths [21]. Even the age when habitat switches occur can be identified by comparing the location of the changes in the ratio of strontium to calcium density along an otolith’s radius with the fish’s age at these Sr:Ca transition zones, as otoliths form age annuli as fish grow older.

Brown trout may spawn in brackish water, as observed from otolith microchemistry [12]. This was verified by a study where spawning by brown trout was observed in a fjord 14 km outside of the River Vosso, Norway [22]. There, the salinity in the spawning gravel was up to 23 psu in the most remote redd, and the offspring survived well according to the authors. Thus, brown trout may not only leave freshwater early in life, but they may also hatch from eggs laid in brackish water. A similar life history with spawning in brackish water is also known from some other salmonids, such as European whitefish (*Coregonus lavaretus*) [23] and pink (*Oncorhynchus gorbuscha*) and chum (*Oncorhynchus keta*) salmon [24,25,26].

Here, we investigated migratory patterns of anadromous brown trout in the Swedish River Emån on the Baltic coast. Brown trout from this river are fast-growing and known for their large size [7,27,28]. We report the age at which the fish migrated from freshwater to saltwater and how many times they returned from the sea to the river, presumably to spawn, based on the Sr:Ca in their otoliths. Based on a previous study in the River Emån [12] and observations of large numbers of salmonid fry at the mouth of the River Emån (unpublished data), we hypothesized that some fish leave the river during their first year of life and that this will be revealed by their otolith microchemistry.

## 2. Materials and Methods

**Figure 1:**
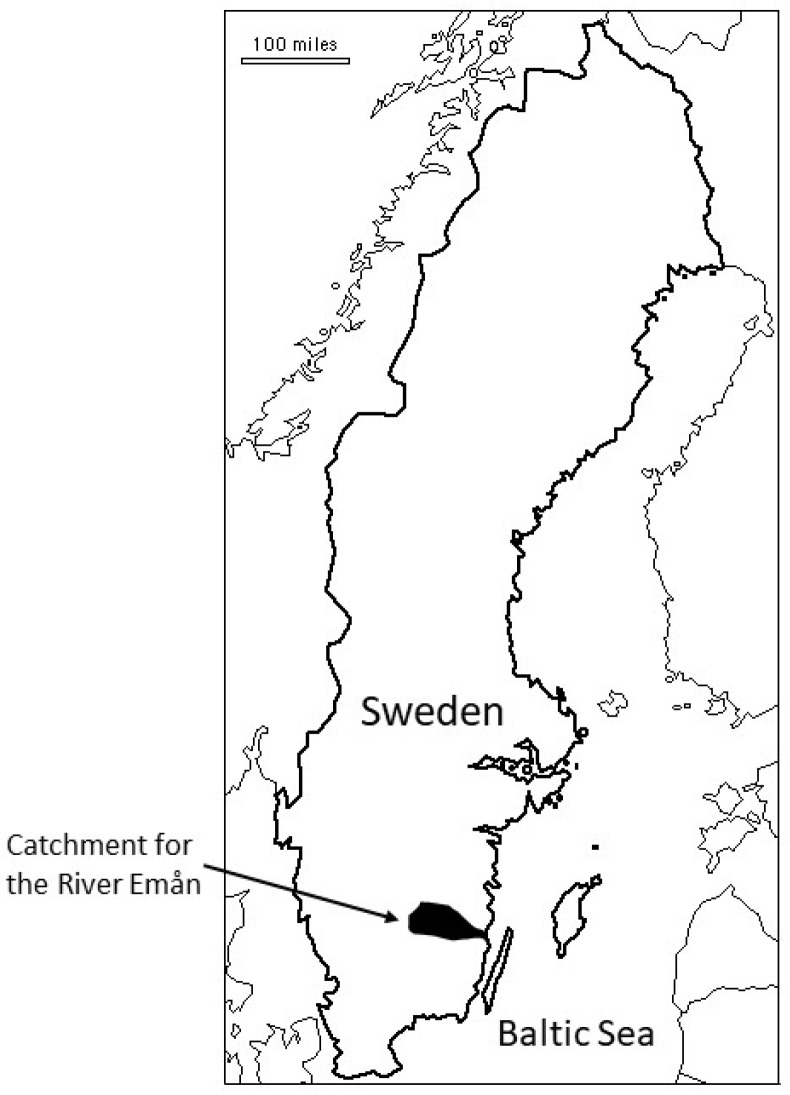
Map of Sweden showing the catchment for the River Emån.

The River Emån, a 229 km long river in southeastern Sweden, has a catchment area of 4470 km^2^ and empties into the Baltic Sea at Em [29] (Figure 1). The River Emån supports 33 fish species and is one of Sweden’s most speciose rivers. Common species in the lower part of the river are Atlantic salmon *Salmo salar*, brown trout, roach *Rutilus rutilus*, Baltic vimba *Vimba vimba*, ide *Leuciscus idus*, and chub *Leuciscus cephalus* [28].

Twenty-three anadromous brown trout, 1.2–9.7 kg in total mass and 47–93 cm in total length, were sampled in the River Emån in 2007 and 2008 (Table 1). The fish were caught by fly fishing and angling by sport fishers from shore and from boats. Seventeen individuals were captured in the river near the river outlet at Em. The remaining six were caught approximately 5 km upstream at Emsfors. From each fish, two sagittal otoliths were removed from the fish for analysis.

Otolith microchemistry was analyzed by the Department of Nuclear Physics, University of Lund, using nuclear microscopy combined with proton-induced X-ray emission analysis, also referred to as PIXE analysis [30,31], using the methodology described by [12]. Measurements of strontium and calcium were made along the otolith’s diameter, every 6 microns from one edge to the other edge through the center of each otolith, representing a so-called life history transect or timeline [32]. The ratio of strontium to calcium (Sr:Ca) was used in this study as a proxy for salinity, where a high ratio indicates that a fish was in saltwater and a low ratio indicates that a fish was in freshwater. A previous laboratory study using water from the Baltic Sea (tested salinity of 6.74‰) verified that differences in Sr:Ca reflected differences in salinity [12]. The study also presented evidence that Sr:Ca varies depending on the salinity that the fish experienced. The authors found higher Sr:Ca values in wild brown trout from the Swedish west coast (where salinities ranged from 20 to 25‰) than in laboratory-raised brown trout that were subjected to an average salinity of 6.74‰. Photos were taken to identify where high and low concentrations of Sr and Ca were found along the otoliths. The fish were aged using a Leica MZ 8 microscope (Leica Microsystems, Bromma, Sweden) with transmitted light projected onto a monitor [33]. In addition to aging the fish, the distance between annuli was measured along the same transect as the elemental composition was measured. By comparing the relative distance of each annulus with the same proportion of points from the elemental analysis, we related changes in elemental composition with age.

We examined otolith maps for strontium to identify transitions between saltwater and freshwater. In addition, plots of Sr:Ca against distance from the otolith’s center were made for each fish. For the Sr:Ca plots, we looked for rapid changes in Sr:Ca as such changes were expected to indicate transitions between freshwater and saltwater, with high ratio values indicating saltwater and low values indicating freshwater. Together, the maps and plots were used to determine when the brown trout left freshwater and swam into the Baltic Sea and to identify the number of return visits to freshwater. The relationships among weight, respective age, and the length of time the brown trout spent in freshwater before moving to the sea (<1 yr; c. 1 yr; >1 yr but <2 yr; ≥2 yrs) and the number of return visits the fish made to freshwater were analyzed. This was performed using a Spearman rank correlation test as data for visits to freshwater were skewed. The weights of four fish were only available as ranges, and in these cases, averages were taken. 

## 3. Results

**Figure 2:**
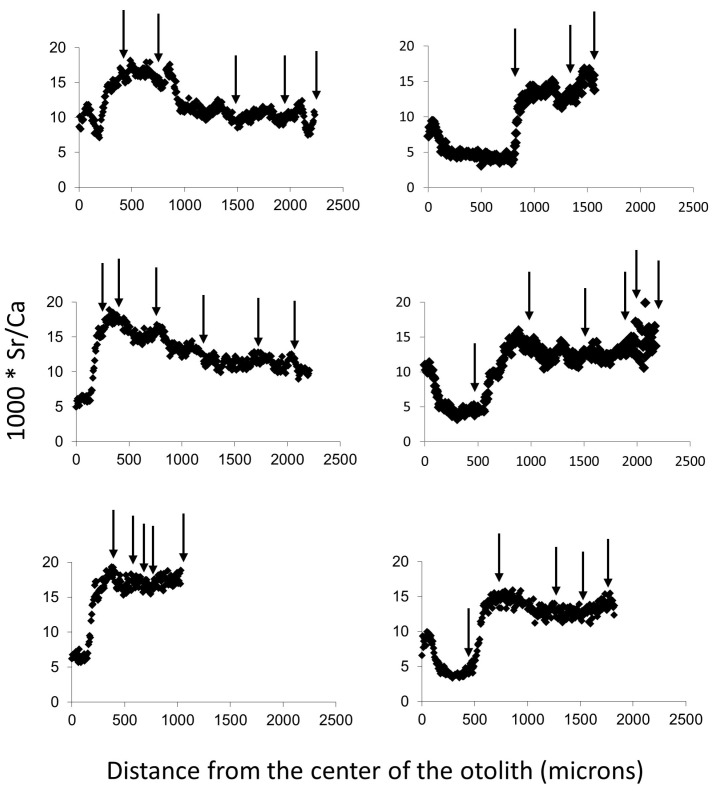
The ratio of Sr to Ca at different distances from the center of the otolith for the six brown trout (*Salmo trutta*) that spent one year or less after hatching in freshwater before migrating to the sea (trout were 3–6 years of age). Arrows indicate the approximate location of annuli. It should also be noted that one can see the maternal saltwater influence on otolith microchemistry, the so-called “mother peaks” (sensu [14]) for four of the fish (4 panels: the top two panels and the three right-hand panels). Note that the three left-hand panels show the brown trout that spent < 1 yr in freshwater before migrating to the sea.

The 23 anadromous brown trout averaged 68 ± 12 cm (SD) in length, 3.6 ± 2.1 kg in wet mass, and 4.4 ± 1.0 years in age (range 3–6 years). The number of females and males was similar, with 10 (43%) believed to be males and 13 (57%) believed to be females (5 males and 8 females when identification of sex was certain; Table 1). Based on the Sr:Ca ratio, 26% of the fish had emigrated to the sea by the age of 1. Three of the fish (13%) spent less than a year in freshwater after hatching (Figure 2). The other three were approximately one year old when they migrated into the Baltic Sea. Forty-eight percent migrated between one and two years of age. The remaining 26% spent more than 2 years in freshwater before migrating to the sea.

**Figure 3:**
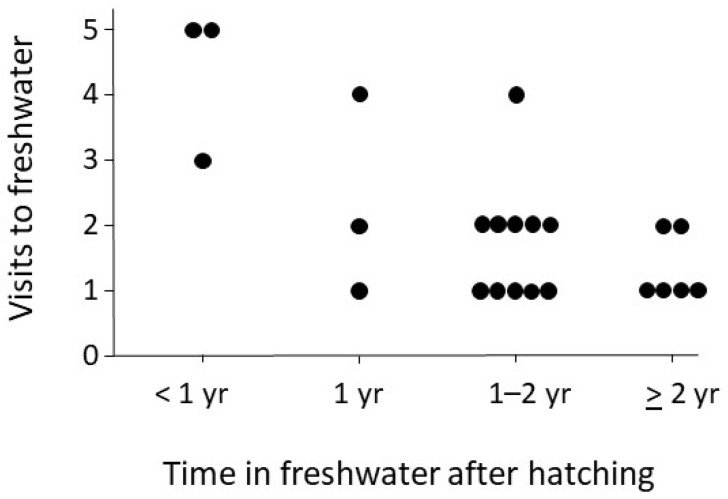
Relationship between the time brown trout (*Salmo trutta*) spent in freshwater after hatching (until the trout migrate to the Baltic Sea) and the number of return visits to freshwater. Note that brown trout that spent the same amount of time in freshwater after hatching (<1 yr, ca. 1 yr, 1–2 yrs, ≥2 yrs) are arranged in rows so that all data points can be seen. N = 23.

**Figure 4:**
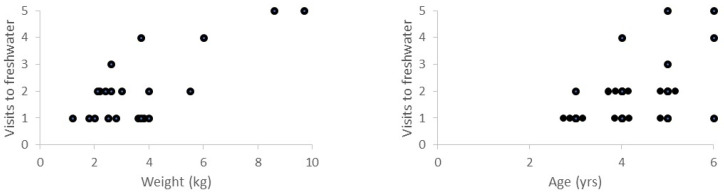
Relationship between weight (kg) and age (yrs) at capture of brown trout (*Salmo trutta*) and the number of return visits to freshwater. Note that brown trout of the same age (right-hand panel) are arranged in rows so that all data points can be seen. N = 23.

On average, the brown trout returned to freshwater 2.0 ± 1.3 times. There was a negative relationship between the length of time the brown trout spent in freshwater before moving to the sea (<1 yr, c. 1 yr, 1–2 yrs, ≥2 yrs) and the number of return visits the fish made to freshwater for spawning (Spearman correlation: rho = −0.555, *p* = 0.006, N = 23; Figure 3). There was also a positive relationship between the weight of the brown trout at capture and the number of return visits to freshwater (Spearman correlation for weight: rho = 0.435 = 0.038, N = 23; Figure 4) and for the age of the brown trout at capture and the number of return visits to freshwater (Spearman correlation for weight: rho = 0.439, *p* = 0.036, N = 23). Weight and age at capture, respectively, were not related to the time the fish spent in freshwater after hatching (Spearman correlation for weight: rho = −0.229, *p* = 0.294, N = 23; Spearman correlation for age: rho = −0.321, *p* = 0.136, N = 23).

## 4. Discussion

Anadromous brown trout in this study spent from less than one to more than two years in freshwater before migrating to the sea. This pattern of freshwater residency is similar to or younger than the 1–3 years reported from other southern Scandinavian rivers [34,35]. However, in an earlier pilot study from the River Emån, the authors reported that five of seven (71%) brown trout that had been sampled had spent less than a year in the river [12]. A similar early emigration was reported from streams on the island of Gotland, Sweden [36], and the Ermespu Stream on Saaremaa Island, Estonia, which drains into Tagalaht Bay of the Baltic Sea [13]. Landergren [11] suggested that individuals who moved very early to the sea were probably not able to defend a territory in freshwater, or they moved to the sea because of drought and habitat loss. The River Emån, which is the largest river in southeastern Sweden, has no summer drought. Thus, a lack of water in the stream is not a plausible explanation for why young brown trout emigrate from this river. Instead, they may leave because of intraspecific competition, difficulty in defending territories in the river, or a lack of proper food [11,37]. Feeding opportunities are assumed to be more abundant at sea than in freshwater at northern latitudes, and these abundant food resources at sea are believed to have led to the evolution of diadromy [38]. Furthermore, fry of brown trout may survive well in brackish water. One study reported that brown trout fry at 3–4 months of age had higher specific growth rates, more effective food conversion ratios, and higher energy absorption efficiencies in brackish water than in freshwater at salinities between 3 and 9 psu [39]. Thus, the brackish water found in the Baltic Sea may be a suitable habitat for young brown trout. Further studies in other geographical regions are needed to determine the generality of the situation observed in the Baltic Sea, i.e., if early migration is related to the availability of brackish water.

Slow-growing fish that are unable to defend a territory are not the only fish that emigrate early from their natal rivers. Forseth et al. [40] studied age at outward migration in inlets to Lake Femund, Norway, and maintained that fast-growing individuals emigrated younger and at a smaller size than slow-growing individuals (but see [41]). A similar finding was reported in a study on anadromous brown trout in Norway [42]. Forseth et al. [40] found that the metabolic demands of fast-growing brown trout were much higher than for those of slow-growing conspecifics, and they were, therefore, sooner constrained by resources in their natal habitat. While the study by Forseth et al. [40] was not based on fry, constrained feeding opportunities relative to their demands and what is present in their alternative feeding habitat may also induce outmigration from the nursery habitat.

According to Werner and Gilliam [43], an optimal habitat is one where mortality risk (µ) divided by growth rate (g) is minimized, and an animal should shift habitats when µ/g in its present habitat is higher than it would be in the alternative habitat. Predation pressure is probably higher in the Baltic Sea than in the River Emån (cf. [44]), making the former a riskier habitat. Therefore, it is an open question as to which habitat is best for the young fish, the river or the sea. Individual fish will not know their mortality risk in alternative habitats, but they may be genetically adapted so that they leave the nursery when their growth rate, possibly associated with their body size, decreases beyond a certain threshold [45]. This threshold may vary among individuals as their size and rate of growth rate differ, which in turn influences mortality risk. This hypothesis agrees with the findings reported by Forseth et al. [40]. Furthermore, other conditions that may influence habitat choices are personality and metabolic rates, both may be influenced by genetics as well as the early environment of the fish (e.g., [46,47]). Thus, the best option may be for some individuals to migrate early, whereas others stay in the river longer before heading for the sea or remain as freshwater residents. Exploring these ideas should be the subject of future research as our sample size is far too small for a proper analysis of the relationship between early growth and size and between age and size at migration.

To date, all studies describing brown trout fry migrating to the sea have studied streams emptying into the Baltic Sea [12,13,36]. In fact, the study by Limburg et al. [12] reported that brown trout may spend their entire life in the Baltic Sea, despite an apparently low hatching success of eggs in brackish water with a salinity above 4‰ [48]. Apparently, the low salinity of the Baltic, which ranges from approximately 2‰ in the north to 12‰ in the south [12], makes this possible. Nevertheless, in this study, the Sr:Ca in the otoliths indicates that all of the fish hatched in the river and thus spent some time in freshwater, albeit < 1 yr for three of the fish (13%). We also have observational data suggesting that fry leave the river in spring. In 2007, we captured thousands of salmonid fry (brown trout and/or Atlantic salmon) in a rotary screw trap placed at the mouth of the River Emån.

It is not unusual for fry and parr of the various species of *Salmo* and *Oncorhynchus* to rear in brackish water. One of the earliest observations of this was published by Huntsman [49]. He wrote that young parr of Atlantic salmon (*Salmo salar*) are known to descend from the spawning area to brackish parts of estuaries, but that they cannot survive in full seawater. Although fry are less well adapted to saltwater than parr, fry of Atlantic salmon regularly move between fresh and brackish water in the Baltic Sea [50]. Similarly, Atlantic salmon parr accompany smolt on their way to the sea, and the authors of one study assumed that brackish water is important for the development of their salt tolerance [51]. The fact that the salinity of water influences the salt tolerance of salmonid fry was verified in experiments with pink salmon (*Oncorhynchus gorbuscha*) fry [52]. A life history strategy where fry rear in brackish water is known from many *Oncorhynchus* species, such as masu salmon (*Oncorhynchus masou*) [53], coho salmon (*Oncorhynchus kisutch*), chinook salmon (*Oncorhynchus tshawytscha*) [54], rainbow trout [55], and sockeye salmon (*Oncorhynchus nerka*) [56], and fry of rainbow trout (*Oncorhynchus mykiss*) have been shown to grow better at salinities between 5 and 10 psu than at higher or lower salinities [57]. A life history strategy where fry rear in brackish water may very well hold for other salmonid species. Indeed, even the offspring of a freshwater resident population of Arctic charr *Salvelinus alpinus* were found to spread among rivers by moving through full seawater, showing that freshwater resident charr tolerate full seawater for a period of time [58].

It is unclear what proportion of the brown trout from the River Emån migrate as fry. Based on our study of adult spawners, 13% of the fish spent less than one year in freshwater, which is slightly higher than the previously reported value (8.5%) for precocious migrants that recruited to coastal and spawning stocks [12]. In addition, 27% of the brown trout left Ar Brook, Gotland, during their first year [12], which is similar to the 26% that spent one year or less in freshwater in the River Emån. Presumably, there was substantial mortality in the Baltic Sea, and we do not know the percentage of the population in the River Emån that migrated as fry.

Our data show that the number of return visits to the River Emån was related to weight and age at capture. Brown trout, being iteroparous, made as many as five return visits to the River Emån for spawning, with an average of two times per fish. This result resembles that of a study in the River Emån, where the authors reported variation in the number of returns to freshwater based on tagged fish, with most fish making two visits, although up to four visits were observed [59]. Elsewhere, it has been found that anadromous brown trout spawn more times in long than short rivers, with more than 70% repeat spawners in the longest streams [34]. Of particular note for our study was the fact that those fish that spent less than one year in freshwater after hatching made more return visits to freshwater than brown trout that spent more time in freshwater, and this was independent of age at capture. This indicates that there may be a trade-off between mortality risk and feeding opportunities in freshwater versus the sea. Moreover, the brown trout that spent substantial time in freshwater before migrating for the first time may have been better adapted to life in the river than in the sea, as suggested for Atlantic salmon *Salmo salar* [60].

Thus, young brown trout may extensively feed in brackish water, although the ecological significance of this is not yet clear [13]. The fact that various life stages of brown trout can live and feed in a variety of habitats suggests that their use is important for the persistence of populations. In the Baltic Sea, we provided evidence suggesting that from very early on, large sea trout arising from fry resided in brackish water. Furthermore, experimental evidence has shown that trout grow faster in brackish water than in freshwater [39]. By including brackish water, the fry may in effect be augmenting fish production, as freshwater rearing habitat is usually assumed to limit the production capacity of brown trout populations [37]. We assume that rearing in brackish water is an active life history choice of the individuals, and this phenotypically plastic behavior expands their habitat and maximizes production. At present, salmonids are threatened by habitat degradation and climate change. The presence of variable habitat choices may thus improve the viability of populations [61].

For fishery managers, the dispersal behavior of salmonids makes abundance estimates difficult. One may easily underestimate the abundance of young fish, as the fraction of fry that enters brackish water is difficult to estimate, and adults may return to the home stream/river for spawning even if they left freshwater as parr and not as smolts [62].

The degree to which anadromous brown trout stray to non-natal streams is highly variable, but some of the largest straying estimates reported are from the Baltic region [63]. This may be related to fry emigrating from natal rivers. If so, imprinting by the fry may be weaker than for those fish that leave as smolts. Alternatively, fish that leave streams as fry may stay at sea longer before returning to freshwater for spawning and, consequently, they may have a greater probability of “forgetting” the location of their origin and returning to the “wrong” river (cf. [64]).

In summary, the present study shows that there is a large variation in the length of time anadromous brown trout spend in freshwater before migrating to the Baltic Sea. Of particular note was the fact that some brown trout migrated from the River Emån into the Baltic Sea during their first year of life and that this was not related to drought as earlier hypothesized; instead, we assume that this may be related to the inability of the fish to feed sufficiently in the river. By extending their area of fry production into brackish water, they may also increase their persistence in a situation with ongoing climate warming and habitat degradation of freshwaters. If so, conservation of natal and juvenile habitats under these premises and threats may require managers to evaluate the situation for brown trout in both freshwater and brackish habitats. This phenotypically plastic habitat use from early life is not yet well understood, and studies of the mortality of fry and older parr will shed further light on how this strategy influences the production of brown trout populations.

## 5. Conclusions

By analyzing the ratio of strontium to calcium in otoliths, we described the use of freshwater and marine environments by anadromous brown trout that spawn in the Swedish River Emån. We observed a large variation in the length of time the brown trout spent in the River Emån before migrating to the Baltic Sea and an inverse relationship between the time spent in freshwater after hatching and the number of visits to freshwater. As expected, most brown trout (74%) in the River Emån migrated to the sea after reaching one or more years of age. Less expected was the fact that some brown trout (13%) migrated from the River Emån to the Baltic Sea during their first year of life, which does not follow the classical pattern in which brown trout spend one or more years in freshwater before migrating to the sea. This new knowledge from another river flowing into the Baltic Sea improves our current knowledge about life history variation in Baltic Sea brown trout. The cause for precocial migration in the River Emån is not known, but our results from this permanently flowing river do not support the idea proposed for other Baltic Sea populations, where the risk of drought has been suggested to be the cause. While drought may be a driver of precocial migration, there are other potential drivers as well. Based on our study in the River Emån, we hypothesize that emigration may be induced when fry experience constrained feeding opportunities relative to their energetic demands [40]. Future studies are needed to explore what drives precocial migration and if it is context-dependent.

## Figures and Tables

**Table 1:** Individual data for the 23 brown trout (*Salmo trutta*) obtained from sport fishers and used in this study. Note that the date of capture was not always recorded, and the sex of many individuals is uncertain. Lengths and weights of some individuals were not recorded or were estimated.

Captured at	Capture Date	Sex	Age (yr)	Length (cm)	Weight (kg)
Em	2007-09-17	male	5+	93	9.7
Em	2008-09-15	male	4+	84	6.0
Em	xxxx-03-05	female?	4	64	2.2
Em	xxxx-03-21	female	5	68	2.6
Em	2008-08-23	female	3+	72	3.8
Em	2008-08-24	female	3+	unknown	2.8
Em	2007-09-10	male	4+	58	2.1
Em	xxxx-04-26	female	4	66	3.0
Em	2008-03-15	female	5	61	2.6
Em	unknown	male?	4+	unknown	c. 2–3
Em	2008-04-20	male	4	62	2.4
Em	2008-03-18	female	6	73	3.7
Em	2008-09-21	male	5+	unknown	5.5
Em	unknown	female?	4+	unknown	c. 2–3
Em	2008-03-17	female	5	unknown	3.6
Em	2008-09-24	female?	3+	47	1.2
Em	2008-08-01	female	6+	85	8.6
Emsfors	2008-03-20	male?	3	55	1.8
Emsfors	2008-04-06	male?	5	68	3.7
Emsfors	2008-04-13	male?	4	61	2.0
Emsfors	Spring 2008	male?	5	unknown	c. 3–5
Emsfors	Spring 2008	female?	6	unknown	c. 3–5
Emsfors	Spring 2008	female?	3	unknown	c. 2

## Data Availability

The data that support the findings of this study are available from the corresponding author upon reasonable request.
